# Reciprocal Relations Between Conflicted Student-teacher Relationship and Children’s Behavior Problems: Within-person Analyses from Norway and the USA

**DOI:** 10.1007/s10802-022-00968-4

**Published:** 2022-10-27

**Authors:** Silje Merethe Husby, Věra Skalická, Zhi Li, Jay Belsky, Lars Wichstrøm

**Affiliations:** 1 Department of Psychology, Norwegian University of Science and Technology, Trondheim, Norway; 2 Department of Child and Adolescent Psychiatry, St. Olav’s Hospital, Trondheim, Norway; 3 School of Psychological and Cognitive Sciences and Beijing Key Laboratory of Behavior and Mental Health, Peking University, Beijing, China; 4 Department of Human Ecology, University of California, Davis, USA

**Keywords:** Behavior problems, Cross-national, Longitudinal, Random intercept, Student-teacher relationship, Within-person

## Abstract

Current evidence suggests that conflicted student-teacher relationships may increase behavior problems in children and vice-versa, but this may be due to confounding. We therefore analyzed their relation applying a within-person approach that adjusts for all time-invariant confounding effects, involving samples from Norway (n = 964, 50.9% females) and the USA (n = 1,150, 48.3% females) followed from age 4–12 years with similar measures. Increased parent-reported behavior problems forecasted increased student-teacher conflict to a similar extent in both countries *(β =* 0.07, *p = .*010), whereas teacher-reported behavior problems predicted increased student-teacher conflict more strongly in Norway (*β* = 0.14, *p* = .001) than in the US (*β* = 0.08, *p* = .050). Increased teacher-child conflict also predicted increased parent-reported *(β =* 0.07, *p = .*010), but not teacher-reported, behavior problems in both countries. Findings underscore the reciprocal relation between behavior problems and a conflictual student-teacher relationship.

**Supplementary Information:**

The online version contains supplementary material available at 10.1007/s10802-022-00968-4.

Scholars contend that a good student-teacher relationship may prevent mental health problems in children, whereas a problematic relationship may increase the risk of developing such problems (e.g., Silver et al., 2005). These claims are supported by observational research, both cross-sectional and longitudinal in design, and with particular reference to student-teacher conflict and child behavior problems (e.g., Pianta & Stuhlman 2004; Silver et al., 2005). There are also theoretical reasons to expect evocative child effects, and evidence from diverse samples indicates that this may be the the case (Nurmi, 2012). In sum, existing prospective studies converge in suggesting a reciprocal relation between student-teacher conflict and behavior problems. If these associations reflect student-teacher conflict being part of the etiology of behavior problems, preventative measures and treatment efforts targeting such conflicts should be developed and evaluated for efficacy. However, if such findings are an artifact of underlying confounding factors, such interventions are unlikely to prove effective. We therefore test whether prospective and reciprocal associations between student-teacher conflict and children’s behavior problems remain when all unobserved time-invariant confounding effects (e.g., genetics, gender) are adjusted for, using a within-person approach.

## Why Teachers Matter for Students and Students Matter for Teachers

As children spend a substantial amount of their weekday hours in school, the school context, including their relationships with teachers, is presumed to contribute significantly to their development (Baker et al., 2008; O’Connor et al., 2011). Indeed, these relationships may be particularly important for children with externalizing behavior problems, as they may be in especial need for close and supportive relationships with sensitive adults in order to develop adaptive emotional and social skills and competences (e.g., Baker et al., 2008; Sabol & Pianta, 2012; Silver et al., 2005). Unfortunately, children displaying externalizing behavior are more likely than others to experience harsh and critical interactions with teachers, and be subject to teaching that is less warm, nurturing and responsive (Hughes et al., 1999).

Disruptive behavior creates a disturbance in class settings, making teacher interventions necessary (Birch & Ladd, 1998). As with coercive cycles of parent-child interaction, some teachers may respond to problematic student behavior with ineffective, inconsistent, and harsh classroom management tactics (Hughes et al., 1999), thereby engendering a conflictual student-teacher relationship. Consistent with this claim is evidence indicating that externalizing behavior at one point is predictive of a more conflictual relationship with teachers later on (e.g., Birch & Ladd 1998; Jerome et al., 2009). This has been conceptualized as a *child-driven model* (Mejia & Hoglund, 2016).

A relationship characterized by high conflict may also promote later disruptive behavior, reflected in findings linking student-teacher conflict with later externalizing problems (Pianta & Stuhlman, 2004; Silver et al., 2005). In this case, the relationship between behavior problems and conflict is viewed as a *relationship-driven model* (Mejia & Hoglund, 2016).

There is also the possibility that externalizing behavior and student-teacher conflict may mutually reinforce each other over time, consistent with a *transactional model* (Mejia & Hoglund, 2016). Support for such a reciprocal relation can be found in studies chronicling externalizing behavior and conflict mutually predicting each other over time (Roorda & Koomen, 2021; Roorda et al., 2014; Skalicka, Belsky, et al., 2015; Skalicka, Stenseng, et al., 2015).

## Why Teachers and Students May Not Matter for Each Other

Despite the evidence and causal processes just considered, it remains the case that teachers’ impact on the development of children’s behavior problems may have, in some respects, been inflated in prior research. Longitudinal studies have identified a range of predictors of behavioral problems, no doubt due to the fact that the etiology of disruptive behavior problems is multifaceted. These include psychological and biological characteristics of the child, such as temperament (e.g., negative emotionality (Wichstrøm et al., 2018); effortful control (Atherton et al., 2019; Wichstrøm et al., 2018); inattention (Bellanti et al., 2000); language disorders (Menting et al., 2011); executive functions (Hobson et al., 2011); physiology (e.g., heart-rate variability and skin conductance (Fanti et al., 2019); brain abnormalities (Thijssen et al., 2015); and genetics (Loeber et al., 2009). Also influential are features of the family, including parenting (Loeber et al., 2009), family structure (Rowe et al., 2002), and socioeconomic circumstances (Bradley & Corwyn, 2002; Hill, 2002). As children grow older, peer relationships also become a source of influence via processes of, for example, rejection and association with deviant peers (Hill, 2002). Factors related to pregnancy have also been tied to the development of behavior problems, perhaps most especially prenatal exposure to drugs, alcohol or tobacco (D’Onofrio et al., 2007), as well as maternal stress (O’Connor et al., 2002). Just as notably, the interactions among any of the effects of these multiple determinants likely contribute to the etiology of child externalizing problems, thus helping to explain why some exposed children develop behavior problems whereas other do not (Loeber et al., 2009). In sum, the myriad of identified predictors of behavior problems outside of the school suggests that conflict with the teacher is unlikely to prove decisive for a child to develop behavior problems.

## Unobserved Confounding

Collectively, the observations just noted also raise the issue of unobserved confounding when seeking to illuminate reciprocal effects linking student-teacher relationships and externalizing problems. To illustrate, high levels of negative emotionality and limited effortful control have been found to predict behavior problems (Wichstrøm et al., 2018), as well as undermine student-teacher relationships–beyond what may be caused by prior, and even covaried, behavior problems, in efforts to predict change in problems over time (Rudasill et al., 2010). Problematical parenting is another potential source of confounding, given its links to problem behavior (Miner & Clarke-Stewart, 2008) and difficult teacher-child relationships, one that is not entirely discounted by controlling for prior behavior problems (Hygen et al., 2017). From a demographic stance, low socio-economic status is associated with more behavior problems (Bradley & Corwyn, 2002) as well as greater student-teacher conflict (Rudasill et al., 2010). Additionally, genetically informed research indicates that aggressive behavior is substantially heritable (Brendgen et al., 2011; Rhee & Waldman, 2002), quite possibly contributing to a conflicted student-teacher relationship (Brendgen et al., 2011). Although a small-scale twin-study (n = 217) failed to find evidence that common genes explained both conflicted student-teacher relationships and behavior problems (Brendgen et al., 2011), the fact that this is the only genetically informed inquiry addressing this issue raises the possibility that both problem behavior and conflictual student-teacher relationships may be influenced by the same genes. Although investigators have made considerable strides towards more precise estimates of the relations between student-teacher conflict and behavior problems by controlling for many relevant confounders (Sabol & Pianta, 2012), a range of factors not adjusted for can still affect any detected associations.

To be appreciated is that current evidence linking student-teacher conflict and child externalizing problems stems from observational research applying regression-type approaches, including cross-lagged panel models. These approaches produce results which are a mixture of between- and within-person variance. The behavior of students or teachers unknown to a specific child–a between-person effect–cannot be involved in the etiology of behavior problems or student-teacher conflicts of the particular child. Rather, causality can only be inferred from within-person results. Several recent statistical approaches are able to tease apart between-person effects from within-person sources of variance (Usami et al., 2019) and by the same token adjust for *all unmeasured time-invariant confounding effects*.

Despite differences between methods, they converge in doing this by including random intercept latent factors which account for the respective *levels* of the constructs in question (e.g., behavior problems and the student-teacher relationship) during the study period. This random intercept then captures what causes one child’s overall levels of the constructs (i.e., a time-invariant effect), and these differences in levels between children are adjusted for in the analyses. Although results from within-person analysis of student-teacher relationships and behavior problems do not warrant the strongest claims of causality, as time-varying confounding effects can still influence the results (e.g., changes in maternal depression), they can certainly aid in moving observational science closer to providing causal insight. For this reason, we have drawn upon the possibilities within the Random Intercept Cross-lagged Panel Model (RI-CLPM; Hamaker et al., 2015). It affords illumination of within-person effects, measuring each individual’s change from each person’s own average level, thereby moving the study of reciprocal effects of student-teacher relationships and child externalizing problems closer to a causal analysis.

In addition to such methodological advance—and given concerns about the replication of findings (Aarts & al., 2015)—we utilize data from two large community studies, conducted in two countries with significant differences in educational systems and rates of externalizing behavior (Heiervang et al., 2007), Norway and the USA, to investigate reciprocal relations between problematic student behavior and conflictual student-teacher relationships. Based on previous findings, we hypothesize that increased student-teacher conflict at one wave will forecast increased externalizing problems at the next wave, and *vice-versa*, when using a traditional autoregressive cross-lag approach. As shown, we believe that there are both theoretical and methodological reasons to believe that these associations may be smaller in magnitude at the within-person level—i.e. *change* in one person predicting later *change* in that person—than those obtained from traditional cross-lagged models which utilize *both* within and between-person information—i.e., a persons’ *level* relative to others predicting ones’ future *level* relative to others. We therefore remain open to the question of whether these predictions hold when all unmeasured time-invariant confounding effects at the between-person level are adjusted for. Finally, we test whether the identified paths differ by country.

## Methods

### Participants and Procedure

**The Trondheim Early Secure Study (TESS)** consists of members of the 2003 and 2004 birth cohorts in Trondheim, Norway (*N* = 3,456) (Steinsbekk & Wichstrøm, 2018). A letter of invitation along with the Strengths and Difficulties Questionnaire (SDQ) 4–16 version (Goodman et al., 2000) was sent to their homes. The SDQ is a brief and valid screen for emotional, behavioral and social problems in the general population (Sveen et al., 2013). Parents were asked to bring the completed SDQ to their child’s scheduled routine 4-year health check-up at their local well child clinic (*n* = 3,358 attended). Here, parents were informed about TESS using procedures approved by the Regional Committee for Medical and Health Research Ethics Mid-Norway, and written consent to participate was obtained. Parents who were not sufficiently proficient in Norwegian to complete the SDQ screening were excluded (*n* = 176). Of those who were asked to participate (*n* = 3,016), 82.2% consented.

To increase statistical power, children with or likely to develop emotional or behavioral problems were oversampled by dividing children into four strata according to their SDQ scores (cut-offs: 0–4, 5–8, 9–11 and 12–40). Using a random number generator, drawing probabilities to participate increased with increasing SDQ scores. The drawing probability increased with increasing SDQ scores of 0.37, 0.48, 0.70, and 0.89 in the four strata, respectively. Of the 1,250 children randomly drawn, 995 were enrolled at T1 (50.9% female). Most parents were either married (56.3%) or had been cohabitating for > 6 months (32.6%). Mothers were predominantly of Norwegian (93.0%) or Western (2.7%) origin. In all, 58.3% had at least a bachelor’s degree whereas 6.7% had not completed senior high school (13 years of education). Drop-out rate after consent did not differ according to SDQ score [(*t* = 0.17, *df* = 1, *p* =. 86) or gender (χ² = 1.02, *df* = 1, *p* = .31]. The mean age at the first assessment was 4.7 years (SD = 0.30, 49.9% males). Retesting occurred at 6 years (T2): *M*_*age*_ = 6.7 years, *SD* = 0.25; 8 years (T3): *M*_*age*_ = 8.8, *SD* = 0.24; and 10 years (T4): *M*_*age*_ = 10.5 years, *SD* = 0.16, and 12 years (T5) M_age_ = 12.5, SD = 0.67. Overall, 964 participants had information from at least one wave of data collection and comprised the analytical sample.

Attrition analyses showed that parent-rated behavior problems at T1 predicted dropout at T3 (odds ratio (OR) = 1.06, 95% confidence interval (CI) = 1.02–1.11). Teacher-rated problem behavior at T1 and T2 predicted dropout at T5 (OR = 1.05, CI = 1.00-1.09, and OR = 1.05, CI = 1.01–1.09, respectively). Response rates among teachers were 91.8-99.1% at T1 through T5.

**NICHD Study of Early Child Care and Youth Development (NICHD SECCYD)** consists of participants recruited from 24 hospitals from 10 data locations in the USA, during the first 11 months of 1991 (NICHD Early Child Care Research Network, 2005) Participants were selected according to a random sampling plan designed to ensure that the sample reflected the demographic diversity of each site’s catchment area. In all, 8,986 women who gave birth during a selected 24 h interval were screened for eligibility. From those, 1,364 families of healthy newborns were included via a home interview one month after birth.

The mean age in kindergarten was 5.6 years (SD = 0.30). Retesting occurred in first grade (T2): *M*_*age*_ = 7.0 years, *SD* = 0.30; third grade (T3): *M*_*age*_ = 9.0, *SD* = 0.31; fifth grade (T4): *M*_*age*_ = 10.7, *SD* = 0.33 and sixth grade (T5) M_age_ = 11.9, SD = 0.35. A total of 1,150 cases comprised the analytical sample (48.3% female). A total of 77.0% of the mothers were married and mothers had on average 14.2 years of education, 80.0% of the children were white and 12.9% were African American. Family income-to-needs was calculated by dividing the family’s gross annual income by the poverty threshold for each family. The average income-to-needs ratio was 3.59 and 16.9% of the families had a score < 1 (below the poverty threshold).

Dropout at T3 was predicted by teacher-rated problem behavior at T1 (OR = 1.04, CI = 1.00-1.09), and dropout at T5 was predicted by teacher-rated problem behavior at T3 and T4 (OR = 1.06, CI = 1.00-1.11 and OR = 1.06, CI = 1.00-1.12, respectively). Response rates among teachers were 83.9–98.1% at T1 through T5.

## Measures

**The Child Behavior Checklist (CBCL**) **and The Teacher Report Form (TRF**). Behavior problems were reported by parents and teachers on the Externalizing Problems scale of the CBCL and the TRF (Achenbach & Rescorla, 2000). The CBCL is a standardized questionnaire designed to be filled out by parents to describe children’s behavioral and emotional problems. The CBCL 4–18 version was used at all time points in the US sample (Achenbach, 1991). In Norway, the 1.5–5 year version was applied at T1, whereas the 6–18 year version was administered at T2 through T5 (Achenbach & Rescorla, 2000, 2001). Norwegian scores were harmonized with the version applied in the USA by summing only the items matching the earlier version (i.e., 25 items at T1—scale range 0–50, 34 items at T2 and later—scale range 0–68). Correlations between the original version scores and the newly created scores were 0.94 at T1 and 0.93 at later time points (α = 0.83-0.88). Day-care centers and schools were requested to select the teacher who knew the child best. In the US, the 5–18 version (Achenbach, 1991) was used. In Norway, the 1.5-5 year version was administered at T1, and the 6–18 year version at T2-T5 (Achenbach & Rescorla, 2000, 2001). Following the procedure described above, the harmonized scores evinced high correlations with original scores (r = .92-0.97; α = 0.92-0.94).

**The Student-Teacher Relationship Scale Short Form (STRS-SF)** is a short form of the original STRS, a frequently used and validated measure that gauges the teacher-perceived relationship quality with individual children (Pianta, 2001). The STRS-SF consists of 15 items and two factors, closeness and conflict, and has been found to have good psychometric properties in differing cultural contexts (Drugli & Hjemdal, 2013; Tsigilis & Gregoriadis, 2008). In both samples we made use of the 7-item conflict scale (scale range: 7–35) that measures the degree to which a teacher feels that his or her relationship with the student in question is characterized by negativity (Tsigilis & Gregoriadis, 2008) (α = 0.81-0.89). The items are rated on a 5-point scale where higher value represents more conflict.

## Analysis Plan

Potential confounding effects can be divided into *time-varying* and *time-invariant* ones. The former change their impact over the time-period under observation (e.g., schooling, peer relationships), whereas the latter do not (e.g., stable effects of genetics, gender) (Wichstrøm et al., 2018). To distinguish within and between person effects we applied structural equation modeling (SEM) in Mplus 7.4, employing a robust maximum likelihood estimator which yields robust standard errors. As attrition analyses suggested that data were missing at random (MAR), missingness was handled with a full information maximum likelihood (FIML) procedure. Due to the aforementioned stratification of the Norwegian sample, its data were weighted with a factor corresponding to the number of children in the stratum divided by the number of participating children in that stratum.

Analyses were conducted in two steps. First, we examined reciprocal relations between student-teacher conflict and child behavior problems by means of traditional autoregressive cross-lagged analysis in which the level of an earlier-measured predictor vis-à-vis others (‘rank-order’) is used to forecast the level of a later-measured outcome (‘rank order’, without regard for individual change). This will allow us to simultaneously test child-driven, relationship-driven and reciprocal models. Because the magnitude of relations between teacher-rated behavior problems and teacher-rated student-teacher conflict might be inflated by common rater bias, two separate autoregressive cross-lagged models were run, one for teacher and one for parent ratings of behavior problems. Because short-term issues might affect parent- and teacher ratings of children (e.g., mood-of-the-day effects or recent conflicts) that are not likely to influence the rating years later, we expected sleeper effects in stability, which pertains to a delayed change in a dependent variable, bypassing intermediate time points (Cook et al., 1979). All measures were therefore auto-regressed on all preceding measures. Error terms of problem behavior and conflict were allowed to correlate at each time point. The scaled chi-square difference test (Satorra & Bentler, 2001) was applied to test equality of cross-lagged paths, and to test equality between countries in a multigroup analysis.

Second, to control for all unmeasured time-invariant effects we employed RI-CLPM for Norway and USA, respectively. This model extends the autoregressive cross-lagged model by separating variance into a stable between-person part (consisting of two latent random intercepts loading on behavior problems and conflict, respectively) and a within-person part; in so doing, it estimates changes from one’s own mean level in a variable (e.g., conflict) as a function of changes in that variable at the previous measurement point (autoregression) and in the predictor (e.g., problem behavior; a cross-lagged effect). A conceptual model is presented in the Online material (Figure S1). We used the scaled chi-square difference (Satorra & Bentler, 2001) to test (i) whether cross-lagged paths were equal over time in each country and (ii) whether there were differences between the countries in a multigroup comparison applying procedures described by Mulder & Hamaker (2020). Because multi-group testing of path equality is based on comparisons of unstandardized path coefficients, we report unstandardized estimates. Because standardized estimates cannot be constrained to be equal, we report mean values of the respective standardized path coefficients, to enable comparisons with other studies. Even though the standardized estimates will be specific to this sample, we follow general guidelines to interpret effect sizes in RI-CLPM, considering 0.03, 0.07, and 0.12 to represent small, medium and large effects, respectively (Orth et al., 2022). Although the RI-CLPM cannot illuminate which unmeasured time-invariant factors prove influential, it is possible to stratify analyses according to measured time-invariant variables. Because behavioral problems as well as student-teacher conflict are more prevalent in boys than girls, we performed gender specific analyses and compared the cross-lagged paths with the procedure just described.

In Norway, the children came from the same city, and thus some belonged to the same school class, and whether multilevel analyses should be performed or not was therefore considered. There are about 250 elementary school classes in Trondheim. Accordingly, the design effect in grades 1–3 varied between 1.2 and 1.6, which is well below the recommended cut-off of 2.0 for when multilevel analyses should be conducted.

## Results

Higher scores on student-teacher conflict and behavior problems were observed in the USA than in Norway (Table S1, available online), and variance in all three measures was also higher in the USA (Table S2). Even so, behavior problems and student-teacher conflict were both concurrently and prospectively positively correlated in both countries, meaning that more conflict was associated with more problems, with no apparent systematic or major differences in magnitude.

**Cross-lagged Panel Models.** We first ran a pair of CLPM, one for teacher-rated behavior problems and one for parent-rated behavior problems in each country, resulting in four models altogether. Then, the cross-lagged paths in the two countries were compared. Based on the Sattora-Bentler test, all cross-lagged paths between conflict and problem behavior in the traditional autoregressive cross-lagged models were found to be equal across all time points, and also equal across both countries for both parent-reported and teacher-reported behavior problems (Table S3). Thus, they indicated that greater student-teacher conflict at an immediately preceding measurement occasion predicted more externalizing problems, irrespective of whether rated by teachers or parents, by the time of the next measurement occasion; and, reciprocally, that greater externalizing problems at an earlier time of measurement, irrespective of whether rated by teachers or parents, similarly predicted higher teacher-student conflict at the next measurement occasion (Supplementary figures S2 and S3). In sum, a transactional model was supported in the CLPM analyses.

## Random Intercept Cross-lagged Panel Models

**Initial Model Testing: Constraining Paths Across Development and Countries.** To test whether predictions conveyed in the CLPM could be attributed to time-invariant confounding effects at the between-person level, RI-CLPM-analyses were employed. First, we ran separate analyses for teacher-rated and parent-rated behavior problems for each country, respectively. Constraining the cross-lagged paths to be equal over time did not worsen the model fit (Table 1), and all such constrained models in each country proved to evince good fit (Table 1). At the between-person level, random intercepts of conflict and problem behavior were moderately (CBCL) to highly (TRF) correlated and consistent in direction with associations described in the preceding paragraph (Norway: CBCL: *r* = .45 (*p* < .001), TRF: *r* = .84 (*p* < .001); USA: CBCL: *r* = .51 (*p* < .001), TRF: *r* = .97 (*p* < .001)). At the next step, the cross-lagged paths could be constrained to be equal across countries without deteriorating model fit in the parent-reported model, while they could not be in the model involving teacher-reported behavior in which they proved different (Table 1).

**Table 1:** Model fit of the random intercept cross lagged panel models and χ² difference test

Countries	Model Fits					*χ² -* difference test across time	*χ² -* difference test across countries
	*χ²*	RMSEA	SRMR	CFI	TLI		
CBCL Norway	47.93 (27), *p* = .008	0.028	0.041	0.982	0.970	5.78 (6), *p* = .45	
CBCL USA	48.10 (27), *p* = .008	0.026	0.034	0.993	0.989	9.49 (6), *p* = .15	
CBCL Norway and USA	60.16 (27), *p* < .001	0.024	0.036	0.992	0.987		0.65 (2), *p* = .72
TRF Norway	56.85 (27), *p* < .001	0.034	0.049	0.981	0.969	8.49 (6), *p* = .20	
TRF USA	50.74 (27), *p* = .004	0.028	0.051	0.993	0.989	4.08 (6), *p* = .67	
TRF Norway and USA	108.52 (54), *p* < .001	0.031	0.050	0.989	0.981		8.55 (2), *p* = .014

*Note*: RMSEA = Root mean square error of approximation, SRMR = Standardized root mean residual, CFI = Comparative fit index, TLI = Tucker-Lewis index, CBCL = Child behavior checklist, TRF = Teacher report form

**Fig. 1:**
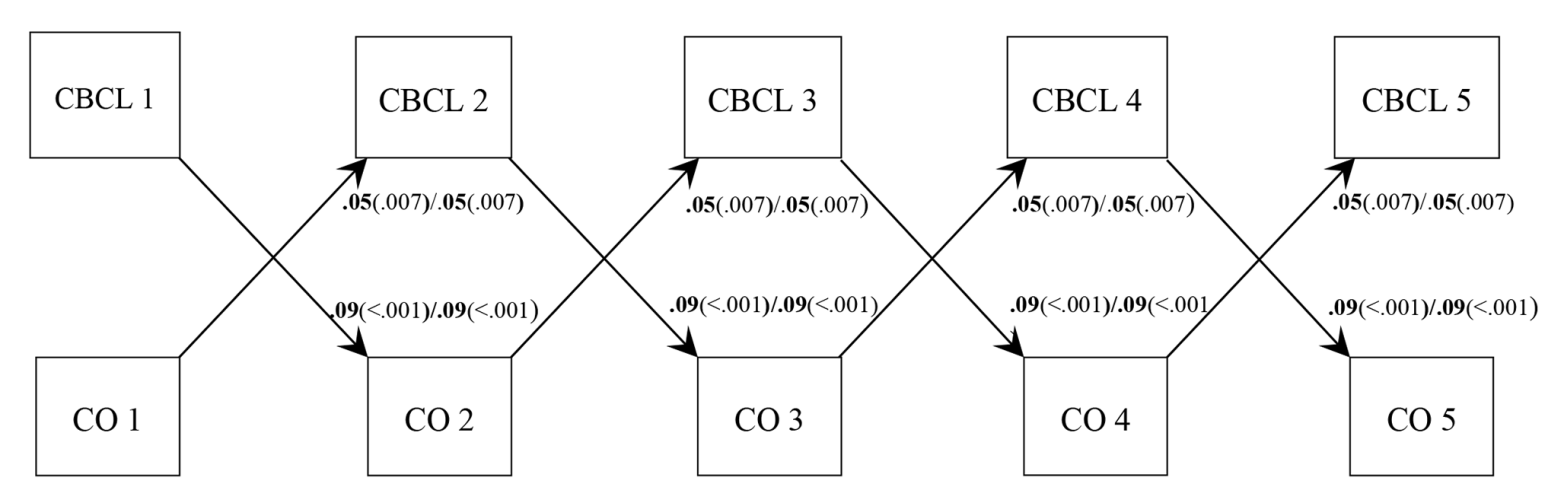
*RI-CLPM showing within-person cross-lagged paths between behavior problems as measured by the CBCL and student-teacher conflict* *Note*: CBCL = Child Behavior Checklist; CO = Student-teacher conflict; Unstandardized path coefficients and p-values, Norway first, USA second. Results from multigroup analyses, where cross-lagged paths were held equal across time and across countries

**Fig. 2:**
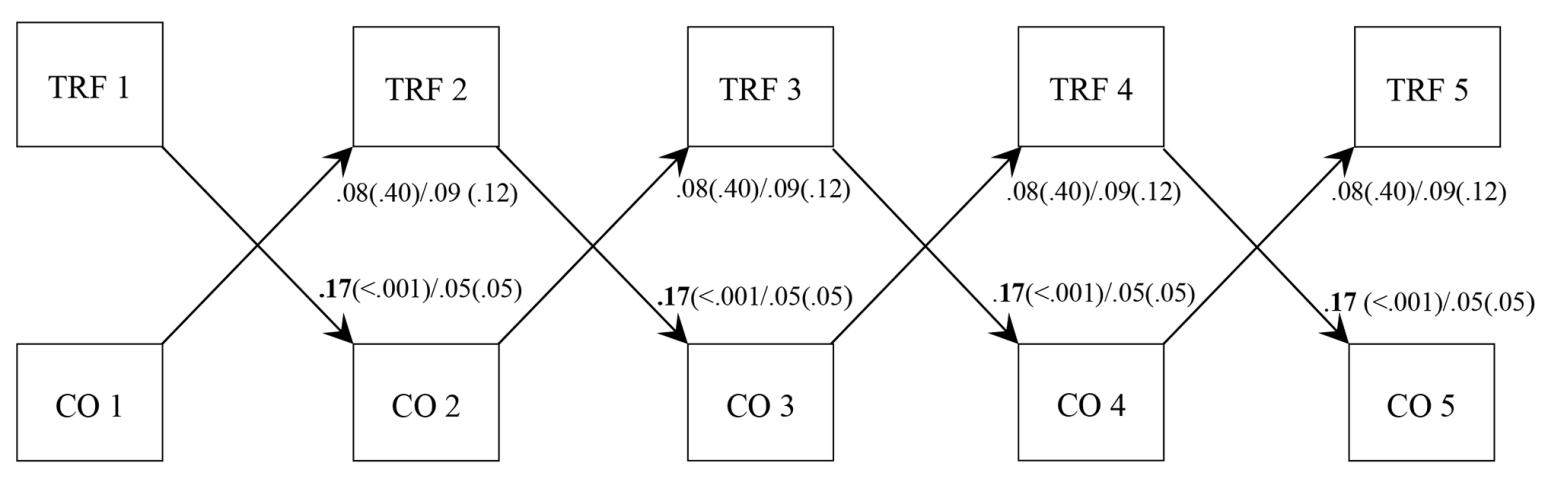
*RI-CLPM showing within-person cross-lagged paths between behavior problems as measured by the TRF and student-teacher conflict* *Note*: TRF = Teacher Report Form; CO = Student-teacher conflict. Unstandardized path coefficients and p-values, Norway first, USA second

**Relationship-driven Model: Student-teacher Conflict Predicting Behavior Problems.** Prospective cross-lagged within-person results (Fig. 1) revealed that increased student-teacher conflict predicted increased parent-rated behavior problems (standardized *β* = 0.06), and the path coefficients did not differ between countries (Table 1). However, increased student-teacher conflict did not forecast increased teacher-rated behavior problems in either country (Fig. 2).

**Child-driven Model: Behavior Problems Predicting Student-teacher Conflict.** Cross-lagged paths involving parent-rated behavior problems predicting later conflict did not differ between countries (Table 1), and a medium effect of increased behavior problems on later increased conflict was identified (*β* = 0.07). In contrast, multigroup analysis of the model involving teacher-rated behavior problems revealed that the cross-lagged paths from increased behavior problems to later increased conflict were stronger in Norway (*β* = 0.14) compared to the estimates in the US (*β* = 0.08; Table 1; Fig. 2). Of note, path coefficients were typically smaller than in the ordinary cross-lagged panel models and medium in magnitude. For example, as can be seen in Fig. 1, when the student-teacher conflict increased with 1 point on the STRS above its average for that relationship, behavior problems increased with 0.05 points on the CBCL scale. One notable exception to these medium effects was the prediction of increased student-teacher conflict from increased teacher-rated behavior problems in Norway.

**Reciprocal Model.** Taken together, the RI-CLPM analyses supported a transactional model with respect to parent rated behavior problems. However, the lack of support for a relationship-driven model when teachers rated behavior problems, prevented support for a transactional model involving teacher ratings of behavior problems.

Gender-specific analyses revealed no differences in magnitudes, with two exceptions. In Norway, increased student-teacher conflict at age 4 predicted more parent rated behavioral problems at age 6 to a stronger degree in boys than in girls, Δχ^2^ = 4.05 (1), p = .04, and increased parent rated behavior problems at age 6 predicted increased student-teacher conflict at age 8, Δχ^2^ = 8.66 (1), p = .003. However, it should be acknowledged that these two significant results emerged after testing 32 paths. Adjusting for the false discovery rate (Benjamini & Hochberg, 1995), these p-values were rendered insignificant, p = .63 and p = .096 respectively. In effect, as the identified cross-lagged effects were the same in girls and boys, gender was not among the time-invariant variables captured by the random intercepts.

## Discussion

Evidence from observational studies suggests that student-teacher conflict contributes to externalizing behavior problems in children and that the reverse is true as well. Yet it remains the case, even when covariates are included in prediction models and prior measurements of the outcome are statistically controlled, that substantial threats to causal inference remain. For this reason, we sought not only to discount time-invariant confounding effects, but also to compare results when these are and are not taken into consideration. The fact that we were not positioned to control for all unmeasured time-varying confounding effects limits, of course, any strong causal inferences that can be drawn from this work.

In the traditional cross-lagged analysis, we found support for a relationship-driven model—more student-teacher conflict at an earlier measurement occasion predicted later behavior problems, as rated by both parents and teachers, in both countries, in line with previous findings (Pianta & Stuhlman, 2004; Roorda & Koomen, 2021; Roorda et al., 2014; Silver et al., 2005). Conversely, a child-driven model was also supported—more behavior problems as rated by both parents and teachers at an earlier measurement occasion, in both countries, predicted more student-teacher conflict at the next measurement occasion. This also aligns with previous findings (Jerome et al., 2009; Mejia & Hoglund, 2016; Pakarinen et al., 2018; Roorda & Koomen, 2021; Roorda et al., 2014), and in sum a transaction model was hence supported irrespective of whether parents or teachers rated the behavior problems.

In the within-person RI-CLPM analysis, that evaluates whether and how change in the predictor forecasts change in the outcome, the support for a relationship-driven model was maintained—increased student-teacher conflict still predicted increased parent-rated behavior problems in both countries when parents assessed the behavior problems. However, when teachers assessed the behavior problems, no effect was seen in either country, perhaps because teachers see children in a more limited setting than parents do, for less time, and only during school hours. As regards a child-driven model, we detected a medium effect of increased parent-rated behavior problems on later increased conflict which was similar in both countries. Such a child-driven effect was also found for teacher-rated behavior problems, and this effect was slightly stronger in Norway than in the US. In sum, a transactional model was supported with respect to parent-rated behavior problems, whereas a child-driven model was supported for teacher-rated behavior problems. Notably, coefficients obtained in the RI-CLPM were generally smaller than in the traditional cross-lagged panel model, the exception being the path between increased teacher-rated behavior problems on later increased student-teacher conflict in Norway. These findings imply that any suggestive causal inferences from traditional approaches risk being overstated and may also be context specific. In the following we advance some possible explanations for the obtained pattern of results.

## A Relationship-driven Model

Although our traditional CLPM results concurred with those of many studies chronicling a predictive effect of student-teacher conflict on later behavior problems as rated by *teachers*, the present effects were not large, and smaller than many of the ones previously reported. However, these larger effects generally stem from smaller sized studies (Birch & Ladd, 1998; Mejia & Hoglund, 2016), whereas studies with sample sizes approaching the ones involved here typically report smaller estimates, comparable to the ones we found (Pianta & Stuhlman, 2004; Roorda & Koomen, 2021). Smaller sample sizes result in larger confidence intervals, implying more uncertainty around the precision of their estimates. With its combination of two large samples from two different countries, the present study has, to the best of our knowledge, the largest and most diverse sample to date, and the medium CLPM estimates therefore line up with previous trends.

In contrast to CLPM results, the effect of student-teacher conflict on *teacher-rated* behavior problems were rendered insignificant in the RI-CLPM—in both countries. This accords with a view that a seeming effect of student-teacher conflict on behavior problems is due to unmeasured time-invariant confounding. Similar to CLPM findings, however, an effect of student-teacher relationship on *parent-rated* behavior problems was retained in the RI-CLPM analysis. Potential reasons for why increased teacher-rated conflict does forecast increased behavior problems as seen by parents, but not teachers, remain unclear.

One possible explanation could be that children experiencing conflict in the student-teacher relationship respond with an increase in problematic behavior at home, in a sort of spillover effect. Previous research indicates that children’s negative experiences at school predict more aversive interactions with parents (Flook & Fuligni, 2008), and higher school stress predicts more aversive family interactions even three years later (Flook & Fuligni, 2008). Thus, it is conceivable that increased student-teacher conflict at school may play out as an increase in externalizing behavior at home. Moreover, parents and teachers usually communicate about the student and their behavior and functioning in school, for example in parent – teacher meetings; and it is possible that should the teacher convey a conflictual or problematic relationship with the child, this may influence parents’ overall impression of their child’s behavior, as a kind of negative halo-effect.

Admittedly, there is no ready explanation for why these effects do not emerge as behavior problems at school. On a methodological note, although the teacher-rated effect fell short of being statistically significant, the effect was almost identical in magnitude to the parent-rated one. Therefore, caution is called for before breathing too much meaning into the parent versus teacher difference.

This report is the first of its kind analyzing data from a large and diverse population, encompassing children, parents and teachers from two different countries with differing school-systems and differing rates of behavior problems. Methodologically, this may have better situated us to provide accurate estimates of the relations between student-teacher conflict and behavior problems. In sum, the findings suggest that previously reported effects of student-teacher conflict on later behavior problems appear to have been inflated due to failure to take into account all time-invariant confounding effects. Regardless of whether effects turned out statistically significant or not, the effects were medium. Even so, these effects emerged at each time point, so effects may accumulate across development. Moreover, the effects appeared at the population level and even medium, averaged effects may prove important for a subset of children. Such moderational effects should be addressed in prospective studies given ever-increasing evidence that children vary in their susceptibility to environmental effects (Belsky & Pluess, 2009).

## A Child-driven Model

An effect of teacher-rated behavior problems on conflict was retained in the RI-CLPM analyses in both countries, being stronger in Norway than in the US. In Norway, students are typically taught by the same teacher from grades 1 to 4 and from grades 5 to 7, making for much greater continuity in the student-teacher relationship than in the US. Hence, there is time to detect a prospective build-up of conflicts in a specific student-teacher relationship when behavior problems increase. Such a build-up may be disrupted in the US by the end of the year, so that—to the extent that previous classes or teachers’ do not forewarn—students and teachers may start with a clean slate each year.

That said, we did find evidence for a predictive effect of change in teacher-rated behavior problems on change in teacher-student conflict in Norway even at those grade-based transitions when teachers change (i.e., from day-care to 1st grade, from elementary to middle school). This is consistent with Brendgen et al.’s (2006) findings that the probability of experiencing teacher verbal abuse proved highly stable even when teachers changed from one year to the next. One possible explanation advanced by Brendgen et al. is that teachers often talk to each other during staff-meetings—and thus that some students develop reputations as troublemakers, deservedly or not. This might help to explain why results proved different in the USA where teacher-teacher communication practices seem likely to be different.

In Norway it is also commonplace for teachers to hold transitional meetings or write transitional reports when children move from day-care (there is no kindergarten in Norway) to school, and when children move from elementary to middle school. This is particularly true in cases in which the child has shown behavioral, social or academic difficulties. The stability of behavior problems was also moderate to high, thus increasing the risk of continuing conflict with a new teacher (Pakarinen et al., 2018).

When child-driven and relationship-driven effects were detected, for both parent and teacher rating of behavior problems and in both Norway and the USA, cross-lagged paths in RI-CLPM did not differ between ages. This lack of developmental differences is notable, as it might have been expected that the importance of the teacher may change over time. Conceivably, the student’s primary teacher may stand out more distinctly as a non-parental socializing agent in preschool and the lower primary grades, thus making conflicts with this teacher especially detrimental. This might seem especially so given that in middle school the student is exposed to several teachers, so that the relationship with a specific teacher becomes less important. But contrary to such an analysis, the concurrent correlation between a negative student-teacher relationship and children’s externalizing problems has been found to be somewhat stronger in the upper primary grades than in kindergarten and the lower primary grades (Lei et al., 2016). Such correlational evidence does not afford much leverage for drawing conclusions about developmental trends in the importance of teachers to students’ externalizing behavior. The present prospective, within-person associations, however, do provide some support for continued importance of the student-teacher relationship at least from preschool age through middle childhood.

In the RI-CLPM, we also detected a reduction in estimates of prospective relations relative to the traditional cross-lagged models. Once again, this would seem to be the result of controlling for time invariant between-person effects from the CLPM estimates so that only actual within-person processes are estimated. Of course, we were not positioned to identify these confounding factors, but there is no shortage of influential suspects at the level of the child, such as genetics (Brendgen et al., 2011), attention problems (Bellanti et al., 2000), temperamental traits (Valiente et al., 2003) and personality traits (Zee et al., 2013); the level of the teacher, such as teacher stress (Yoon, 2002), and classroom management strategies (Korpershoek et al., 2016); or other contextual factors, such as parenting practices (Hygen et al., 2017) and low socio-economic status (Bradley & Corwyn, 2002). This is a task awaiting future research.

## Strengths and Limitations

Despite using prospective and multisource data from two large population studies in countries with differing educational systems, and strong analytical techniques that discount one important set of confounding effects—time-invariant ones—this report is not without limitations. The student-teacher relationship is a two-way street, and teacher-reports could be affected by personal characteristics, appraisals, experiences and expectations on part of the teacher, thereby reflecting teacher-characteristics rather than actual conflict (Thijs & Koomen, 2009). However, many of these biases could be considered more or less time-invariant and thus are adjusted for in the RI-CLPM analyses. Also, we were only able to capture the teacher’s appraisal of the amount of student-teacher conflict, and there is some evidence to indicate that student and teacher ratings of their relationship do not correspond well (Murray et al., 2008). An observational measure might have provided a more unbiased account of conflict-level. Even so, the STRS’ conflict dimension and conflict ratings of external observers have at least moderate agreement (Thijs & Koomen, 2009).

Although Norway and the USA differ in educational systems and behavior problems, they are still Western and affluent countries. Nevertheless, research indicates that teacher’s perceptions, interpretations and expectations regarding children’s behavior are somewhat culture specific, and behaviors that are conducive to the relationship in one culture may actually be associated with higher levels of conflict in another (Gregoriadis & Tsigilis, 2008). Hence, generalization to locales with different educational systems or sociocultural conditions or different ethnicities should be done with utmost care. Subgroups of children—such as those with disabilities or those doing poorly academically (Hamre et al., 2008; Murray & Murray, 2004)—may also be differentially at risk for, and sensitive to, conflict in the student-teacher relationship (McGrath & Van Bergen, 2015). Hence, although we find medium to small overall effects, for some children—or teachers—the conflict-behavior problems link may be stronger than is portrayed here. It is also possible, given evidence of differential susceptibility to many environmental effects (Belsky & Pluess, 2009), that the general or average effect sizes evaluated herein mask meaningful variation in the degree to which both individual teachers and children proved susceptible to relational processes at school.

## Conclusion

A large body of research into the teacher-child relationship clearly suggests that this is a relationship of significant developmental importance. Prior observational research has chronicled reciprocal effects linking increased student-teacher conflict and exacerbated behavior problems. Causal interpretations of such associations have led to suggestions that the student-teacher relationship may be a target of intervention to help prevent behavior problems (e.g., O’Connor et al., 2011). Capitalizing on the possibilities of within-person analysis, we find that these anticipated statistical associations are evident in both Norway and the USA, and from preschool or day-care through middle childhood. These findings render support for a reciprocal relationship between a conflictual student-teacher relationship and child behavior problems, as assessed by parents, and from teacher-rated behavior problems to conflicts in the student-teacher relationship. However, even though the effects are smaller than previously portrayed when time-invariant effects were not adjusted for, they still lend some support to the importance of the student-teacher relationship for the development of behavior problems and vice-versa. Future work should seek to determine whether some children prove more susceptible to the effects under consideration, with the same perhaps worth considering in the case of teachers. There are no doubt gains to be made from avoiding student-teacher conflict on many parameters relevant to both student and teacher adjustment and well-being, but the effect on behavior problems is likely smaller than previously assumed.

## Electronic Supplementary Material

Below is the link to the electronic supplementary material.

Supplementary Material 1
