# Supplementary material for: Reciprocal Relations Between Conflicted Student-teacher Relationship and Children’s Behavior Problems: Within-person Analyses from Norway and the USA
Source: Res Child Adolesc Psychopathol. 2022 Oct 27;51(3):331–42. doi: 10.1007/s10802-022-00968-4 (PMC9908624; doi:10.1007/s10802-022-00968-4)
Supplement: Supplementary file 1 — Supplementary Material 1 [file 10802_2022_968_MOESM1_ESM.docx]

Figure S1: Conceptual illustration of the RI-CLPM model


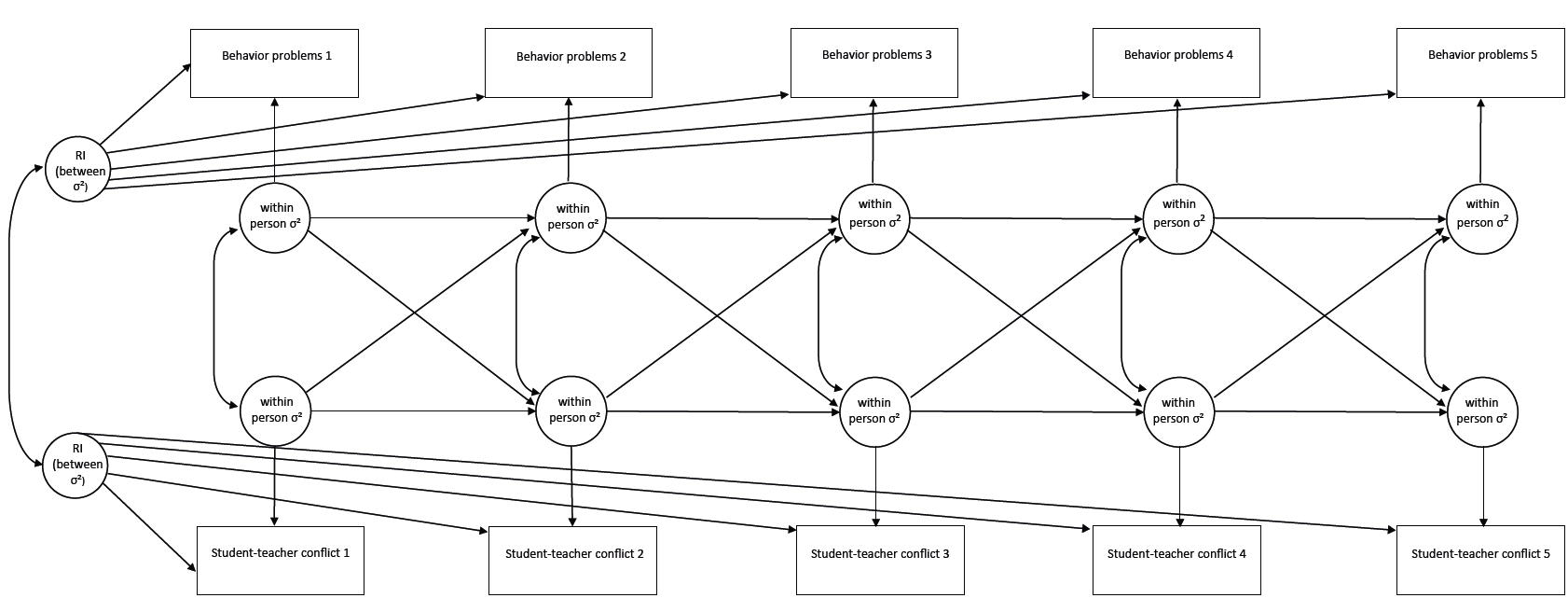


**Table S1**

*Correlations, means and SDs of study variables*

|  |  |  | USA | | | | | | | | | | | | | | | | |
| --- | --- | --- | --- | --- | --- | --- | --- | --- | --- | --- | --- | --- | --- | --- | --- | --- | --- | --- | --- |
| Measu-res | M | SD | CO1 | CO2 | CO3 | CO4 | CO5 | CBCL1 | CBCL2 | CBCL3 | CBCL4 | CBCL5 | TRF1 | TRF2 | TRF3 | TRF4 | TRF5 | M | SD |
| CO1 | 8.81 | 2.99 | - | .40*** | .36*** | .28*** | .22*** | .24*** | .26*** | .24*** | .23*** | .20*** | .81*** | .43*** | .38*** | .30*** | .26*** | 10.60 | 5.36 |
| CO2 | 8.63 | 3.35 | .29*** | - | .48*** | .45*** | .41*** | .24*** | .32*** | .32*** | .26*** | .26*** | .41*** | .79*** | .46*** | .44*** | .44*** | 10.92 | 5.17 |
| CO3 | 8.37 | 3.03 | .32*** | .57*** | - | .47*** | .45*** | .23*** | .33*** | .35*** | .27*** | .26*** | .40*** | .51*** | .82*** | .48*** | .49*** | 11.62 | 6.03 |
| CO4 | 8.52 | 3.38 | .32*** | .50*** | .51*** | - | .54*** | .24*** | .27*** | .31*** | .31*** | .31*** | .30*** | .45*** | .48*** | .80*** | .53*** | 11.44 | 5.74 |
| CO5 | 8.61 | 3.47 | .27*** | .31*** | .44*** | .56*** | - | .22*** | .31*** | .30*** | .32*** | .34*** | .26*** | .43*** | .50*** | .53*** | .78*** | 11.07 | 5.64 |
| CBCL1 | 2.99 | 3.00 | .16*** | .18*** | .22*** | .16*** | .16*** | - | .75*** | .66*** | .62*** | .61*** | .28*** | .27*** | .25*** | .23*** | .22*** | 8.94 | 6.66 |
| CBCL2 | 3.75 | 4.23 | .27*** | .32*** | .30*** | .23*** | .24*** | .60*** | - | .73*** | .69*** | .66*** | .29*** | .33*** | .38*** | .28*** | .32*** | 8.15 | 6.59 |
| CBCL3 | 2.66 | 3.52 | .29*** | .27*** | .32*** | .26*** | .24*** | .50*** | .67*** | - | .73*** | .67*** | .30*** | .33*** | .40*** | .28*** | .27*** | 7.39 | 6.32 |
| CBCL4 | 2.13 | 3.09 | .26*** | .23*** | .27*** | .30*** | .26*** | .50*** | .62*** | .71*** | - | .80*** | .30*** | .31*** | .32*** | .31*** | .34*** | 6.56 | 6.33 |
| CBCL5 | 1.96 | 3.63 | .18*** | .17*** | .26*** | .22*** | .30*** | .38*** | .50*** | .58*** | .80*** | - | .25*** | .27*** | .31*** | .28*** | .34*** | 6.24 | 6.21 |
| TRF1 | 2.75 | 4.38 | .67*** | .34*** | .34*** | .30*** | 21.*** | .18*** | .32*** | .32*** | .26*** | .20*** | - | .48*** | .45*** | .34*** | .31*** | 5.10 | 8.25 |
| TRF2 | 3.76 | 6.41 | .36*** | .75*** | .57*** | .44*** | .36*** | .18*** | .41*** | .34*** | .33*** | .24*** | .46*** | - | .52*** | .49*** | .52*** | 5.63 | 8.17 |
| TRF3 | 3.22 | 6.19 | .35*** | .50*** | .72*** | .47*** | .46*** | .20*** | .40*** | .42*** | .39*** | .26*** | .48*** | .66*** | - | .53*** | .53*** | 6.47 | 9.37 |
| TRF4 | 3.24 | 6.07 | .34*** | .53*** | .57*** | .77*** | .58*** | .18*** | .36*** | .33*** | .40*** | .32*** | .40*** | .58*** | .66*** | - | .59*** | 6.07 | 9.20 |
| TRF5 | 3.06 | 6.04 | .21** | .28*** | .44*** | .48*** | .75*** | .17*** | .29*** | .29*** | .35*** | .39*** | .26*** | .38*** | .57*** | .70*** | - | 5.55 | 9.17 |
|  |  |  | NORWAY | | | | | | | | | | | | | | | | |

*Note:* CO = Conflictual student-teacher relationship as measured by the Student-teacher relationship scale; CBCL = Child behavior problems measured by parent reports in the Child behavior checklist; TRF = Child behavior problems measured by the Teacher report form. Numbers behind the measures indicate data collection wave. * *p* <.05, ** *p* <.01, *** *p* < .001.

**Table S2**

*χ² difference test of equality in means and variance across countries*

|  | *χ² (df)* | *p*-value |
| --- | --- | --- |
| CBCL means | 1726.20 (5) | <.001 |
| CBCL variance | 334.88 (5) | <.001 |
| TRF means | 245.59 (5) | <.001 |
| TRF variance | 135.85 (5) | <.001 |
| Conflict means | 570.96 (5) | <.001 |
| Conflict variance | 163.72 (5) | <.001 |

**Table S3**

*Model fit of the cross-lagged panel models and χ² difference test*

| Countries and measures | *χ² -* difference tests | | Model fits | | | | |
| --- | --- | --- | --- | --- | --- | --- | --- |
|  | Across time | Across countries | *χ²* | RMSEA | SRMR | CFI | TLI |
| CBCL Norway | 12.92 (6), *p* =.04 | 13.18 (8), *p* = .11 | 87.83 (48),  *p* < .001 | .028 | .035 | .990 | .981 |
| CBCL USA | 8.23 (6), *p* = .22 |  |  |  |  |  |  |
| TRF Norway | 12.31 (6), *p* = .06 | 3.41 (2), *p* = .18 | 106.72 (48),  *p* < .001 | .034 | .049 | .986 | .974 |
| TRF USA | 6.40 (6), *p* = .38 |  |  |  |  |  |  |

**Figure S2**

*CLPM showing cross-lagged paths between behavior problems as measured by the CBCL and student-teacher conflict*

CO 5

.**07**(<.001)/.**07**(<.001)

.**07**(<.001)/.**07**(<.001)

.**07**(<.001)/.**07**(<.001)

.**07**(<.001)/.**07**(<.001)

18

vcv cv fxg88 8..18

CBCL 1

CBCL 4

CO 1

CO 4

.**12**(<.001)/.**12**(<.001)

.**12**(<.001)/.**12**(<.001)

18

vcv cv fxg88 8..18

.**12**(<.001)/.**12**(<.001)

18

vcv cv fxg88 8..18

.**12**(<.001)/.**12**(<.001)

CBCL 2

CBCL 3

CO 3

CO 2

CBCL 5

*Note*. CBCL=Child Behavior Checklist; CO=Student-teacher conflict; Unstandardized path coefficients and p-values, Norway first, USA second. Cross-lagged paths were held equal across time and across countries.

**Figure S3**

*CLPM showing cross-lagged paths between behavior problems as measured by the TRF and student-teacher conflict*

.**16**(.001**)**/.**16**(.001)

18

vcv cv fxg88 8..18

CO 5

.**16**(.001**)**/.**16**(.001)

.**16**(.001**)**/.**16**(.001**)**

.**16**(.001**)**/.**16**(.001)

.**16**(.001**)**/.**16**(.001)

18

vcv cv fxg88 8..18

TRF 1

TRF 4

CO 1

CO 4

.**16**(.001**)**/.**16**(.001)

.**16**(.001**)**/.**16**(.001)

18

vcv cv fxg88 8..18

.**16**(.001**)**/.**16**(.001)

18

vcv cv fxg88 8..18

.**16**(.001**)**/.**16**(.001)

TRF 2

TRF 3

CO 3

CO 2

TRF 5

*Note*. TRF=Teacher Report Form; CO=Student-teacher conflict. Unstandardized path coefficients and p-values, Norway first, USA second. Cross-lagged paths were held equal across time and across countries.
